# Morusin suppresses breast cancer cell growth in vitro and in vivo through C/EBPβ and PPARγ mediated lipoapoptosis

**DOI:** 10.1186/s13046-015-0252-4

**Published:** 2015-11-04

**Authors:** Haiyan Li, Qiaoping Wang, Lihua Dong, Chuanlan Liu, Zhen Sun, Ling Gao, Xiujie Wang

**Affiliations:** Laboratory of Experimental Oncology,State Key Laboratory of Biotherapy/ Collaborative Innovation Center for Biotherapy, West China Hospital, West China Clinical Medical School, Sichuan University, Chengdu, 610041 China

**Keywords:** Morusin, Breast cancer, Growth inhibition, Adipogenic differentiation, Apoptosis, Lipoapoptosis

## Abstract

**Background:**

Breast cancer is the most fatal malignant cancer among women, the conventional therapeutic modalities of it are limited. Morusin possesses cytotoxicity against some cancer cells in vitro. The purpose of this study is to test the growth inhibition effect of morusin on human breast cancer growth in vitro and in vivo and to explore the potential mechanism of its action.

**Methods:**

The growth inhibition effect of morusin on human breast cancer cells in vitro and in vivo were tested by cell cytotoxicity, colony formation inhibition, adipogenic differentiation, apoptosis induction, and tumor growth inhibition in vivo assays. The potential molecular mechanisms underlying the growth inhibition effect of morusin on human breast cancer cells in vitro and in vivo were investigated with Western blotting evaluation of expression levels of transcription factors, C/EBPβ and PPARγ, adipogenic and apoptotic proteins in morusin treated breast cancer cells and tumor tissues.

**Results:**

Morusin inhibited breast cancer cells growth in vitro and in vivo; it induced adipogenic differentiation, apoptosis and lipoapoptosis of cancer cells.

**Conclusions:**

Morusin has the potential to inhibit human breast cancer cell growth in vitro and in vivo through C/EBPβ and PPARγ mediated lipoapoptosis.

## Background

Breast cancer is one of the most prevalent cancers and the leading cause of cancer death among women worldwide [1]. Despite the significant advances in breast cancer treatment modalities and improvement of patients’ survival and quality of life in recent decades, its incidence and mortality are increasing steadily, especially in developing countries [1–3]. Currently, the conventional therapeutic strategies such as surgery, radiotherapy, and chemotherapy are limited treatment options for breast cancer. Although breast cancer patients with estrogen receptor positive (ER+) have a better outcome after endocrine therapy, one-third of them are not sensitive to Tamoxifen, and the rest of them have a risk of relapse [4, 5]; The subtype, “Triple Negative Breast Cancer” (TNBC), is more aggressive and resistance to available treatments, there has no available therapeutics for it [6, 7]. Therefore, the identification of effective chemopreventive agents and development of neoadjuvant chemotherapies with alternative strategic options are crucial for ER+ breast cancer and TNBC [8–11].

Previous investigations revealed natural products process anticancer activity and selectivity of anti-cancer agents [12, 13], flavonoids provide a diversity of anticancer compounds which can be used for breast cancer prevention and/or treatment [14].

Morusin is a prenylated flavonoid derived from the root bark of Morusaustralis (Moraceae) [15] and branch bark of Ramulus mori [16], possesses anti-oxidant and anti-inflammatory activities [17]. It exhibited cytotoxicity against some human cancer cells in vitro, including colorectal cancer [15], prostate cancer [17], breast cancer, cervical cancer and liver cancer cells [18, 19], prevents neuronal cells from nitrosative stress-mediated cell death [20], and inhibits the tumor growth of murine hepatocarcinoma in vivo without side effects [11]. Our previous studies showed that morusin inhibited the proliferation and migration of human cervical CSCs through reduction of NF-κBp65 activity and apoptosis induction [21], suppressed glioblastoma stem cell growth in vitro and in vivo through stemness attenuation, adipocyte transdifferentiation and apoptosis induction [22].

In light of these findings, it could be assumed that morusin might serve as a novel therapeutic agent for cancer therapy, But its anticancer efficiency and profile needs to be confirmed further, and the mechanism of action is elusive [17–22]. Therefore, in the present study, we investigated the growth inhibition effect of morusin on human breast cancer cells in vitro and in vivo and characterized its potential mechanism of anticancer activity.

## Methods

### Reagents

DMEM media and fetal bovine serum (FBS) were purchased from Invitrogen (Shanghai, China). Trypsin, 3-(4, 5-dimethylthiazol-2-yl)-2, 5-diphenyltetrazolium bromide (MTT), DMSO and other chemicals and reagents were obtained from Sigma-Aldrich (Shanghai, China). Morusin was purchased from Chengdu Biopurify Phytochemicals Ltd. (Chengdu, China, purity ≥98 % HPLC).

### Cell line and culture

Human normal mammary epithelial cells, MCF-10A, murine breast cancer cells (4 T1 and EMT6) and human breast cancer cells (MCF-7 and MDA-MB-231) were obtained from Shanghai Cell Biology Institute of Chinese Academy of Sciences (Shanghai, China), and were maintained in DMEM medium with 10 % fetal bovine serum, penicillin (100 U/ml) and streptomycin (100 μg/ml) at 37 °C in the presence of 5 % CO_2_.

### Cytotoxicity assay (MTT)

The cytotoxicity of morusin against human normal mammary epithelial cells and murine breast cancer cells (4 T1 and EMT6) and human breast cancer cells (MCF-7 and MDA-MB-231) was tested by modified MTT assay [23]. Briefly, human normal mammary epithelial cells MCF-10A, and breast cancer cells, MCF-7 and MDA-MB-231, (1 × 10^3^/well) were seed in 100 μl of medium/well in 96-well plates. After overnight incubation, the cells were then treated with various concentrations of morusin (1, 2, 4, 6 and 8 μg/ml), each concentration containing 3 wells. After treatment with morusin for 1, 2, 3, 4, and 5 days, 20 μl MTT (pH 4.7) was added to each well, and cultivated for another 4 h, 100 μL of 10 % SDS/0.01 N HCl was added and incubated at 37 °C overnight to dissolve the formazan. Absorbance was measured at 570 nm, the effect of morusin on the viabilities of normal mammary epithelial cells, MCF-10A and breast cancer cells, MCF-7 and MDA-MB-231 were expressed as the % cytoviability, using the following formula: % cytoviability = A_570_ of treated cells/A_570_ of control cells × 100 % [23, 24], Three independent experiments were performed.

### Colony formation inhibition assay

The clonogenic potential of breast cancer cells was determined by seeding 300 human breast cancer cells per well in 6-well plates, the cells were incubated for approximately 24 h, and then treated with 1, 2, and 3 μg/ml of morusin, respectively. After 12 days of incubation, the cells were stained with 0.5 % crystal violet in absolute ethanol and colonies with >50 cells were counted under dissection microscope. Three independent experiments were conducted, each in triplicate.

### DAPI staining apoptotic cells

Apoptotic morphology-nuclear chromatin condensation was examined with DAPI staining. MCF-7 and MDA-MB-231 cells were seeded in 6-well plates. After incubation overnight, cells were treated with different concentrations of morusin (4, 6, 8 μg/ml) for 48 h. Subsequently, cells were harvested, fixed in 4 % paraformaldehyde, treated with 0.25 % Triton X-100 in TBS for 15 min at room temperature and stained with 50 μl DAPI (4 mg/mL, Sigma, Aldrich) for 30 min at room temperature. After washing with PBS, samples were stored in the dark at 4 °C and examined under a fluorescence microscope [25], three independent experiments were conducted.

### Annexin V-FITC/PI double staining apoptotic cells

Detection of apoptotic cells was performed using the Annexin V-FITC/PI apoptosis detection kit (Beyotime Biotech, Shanghai, China) according to manufacturer’s instructions. Briefly, cancer cells treated with 4, 6, and 8 μg/ml of morusin, respectively, for 36 h were washed with PBS and stained simultaneously with FITC-conjugated Annexin V and PI at room temperature for 15 min in the dark. The apoptotic cells were measured using a FACScalibur flow cytometer and Cell Quest Pro software (BD Biosciences, Shanghai, China). Three independent experiments were performed.

### Cell cycle analysis (FCM)

Cell cycle distribution was analyzed by flow cytometry (FCM). Briefly, 1 × 10^6^ cells were harvested from the control and breast cancer cells treated with 4, 6, 8 μg/ml of morusin for 36 h, washed twice with PBS and fixed in 70 % ice-cold ethanol for 1 h. The sample was then concentrated by removing ethanol and treated with 1 % (v/v) Triton X-100 and 0.01 % RNase for 10 min at 37 °C. Cellular DNA was stained with 0.05 % propidium iodide for 20 min at 4 °C in darkness. Cell cycle distribution were analyzed with FCM (Cytomics™ FC500,Beckman Coulter) and MultiCycle software package (Phoenix, USA). All data represents the results from three independent experiments.

### Tumor growth inhibition test in vivo

15 six-week-old female nude mice were inoculated with 2 × 10^6^ human breast cancer cells (MCF-7) subcutaneously. After 5 days of tumor cell inoculation, tumor bearing mice were randomized into three groups, each having five mice. Two treatment group mice were injected with 5 and 10 mg/kg of morusin i.p. three times weekly for 4 weeks, respectively, and the control mice were injected with DMSO. During the experiment, mice were weighted, and tumor volumes were measured weekly using calipers and their volumes were calculated using a standard formula (length × width^2^ × 0.5) [26]. At the end of experiment, the mice were sacrificed by carbon dioxide asphyxiation; tumor masses were dissected, and weighed. The tumor inhibitory rates were calculated using the following formula: tumor inhibitory rate(%) = (mean tumor weight of the control mice − mean tumor weight of the treated mice)÷ mean tumor weight of the control mice × 100 %. The experiment was performed under standard conditions according to the guidelines of the Institutional Animal Care and Use Committee of Sichuan University.

### Oil Red O staining

Breast cancer cells treated with 2, and 4 μg/ml of morusin for 72 h, respectively, the cells were fixed with 100 % methanol, washed in PBS; cryostat sections of the control and morusin treated tumor tissues were prepared regularly. Both the cells and cryostat sections of tumor tissues were stained with Oil Red O stain for 20 min at room temperature [27]. After incubation, slides were differentiated with an 85 % propylene glycol solution for 1 min, rinsed in water, and counterstained with Mayer’s Hematoxylin and observed under microscope.

### Western blot analysis

Both morusin treated breast cancer cells and tumor tissues were lysated with RIPA lysis buffer, centrifuged, the supernatants were collected and quantified with UV spectrophotometer. Samples containing 30 μg protein were mixed with loading buffer (5×), boiled for 5 min, separated by 12 % SDS-PAGE, transferred PVDF membranes using a semi-dry blotting apparatus (Bio-Rad, Hercules, CA, USA), and then blocked in 5 % non-fat milk at RT for 1 h. The PVDF membranes were incubated with primary antibodies of rabbit anti-C/EBP β,rabbit anti-PPARγ, rabbit anti-adipsin D,rabbit anti- perilipin A + B, rabbit anti-Bcl-2, rabbit anti-Bax, rabbit anti-active caspase-3 and rabbit anti-β-actin (Beijing Biosynthesis Biotechnology Co., LTD, Beijing, China) diluted 1:300. Antibody recognition was detected with peroxidase-conjugated goat anti-rabbit IgG (H + L) secondary antibody (Zhongshan Goldenbridge Biotechnology Co., LTD, Beijing, China) used at 1:6000 dilutions, antibody-bound proteins were detected by Chemiluminescent HRP Substrate (Milipore Corporation, Billera, USA) and western blotting analysis system (Universal Hood II, Bio-Rad, USA), and normalized to β-actin and semi-quantified using the ChemiDocTM XRS (Bio-Rad, USA).

### Statistical analysis

The data were expressed as mean ± standard deviation (Mean ± SD). All data were analyzed using the software SPSS V 16.0. Independent sample *t*-test was used to analyze the statistical difference. Statistical significance was defined as *p* < 0.05 for all tests.

## Results

### Morusin inhibits murine and human breast cancer cell proliferation

**Fig. 1:**
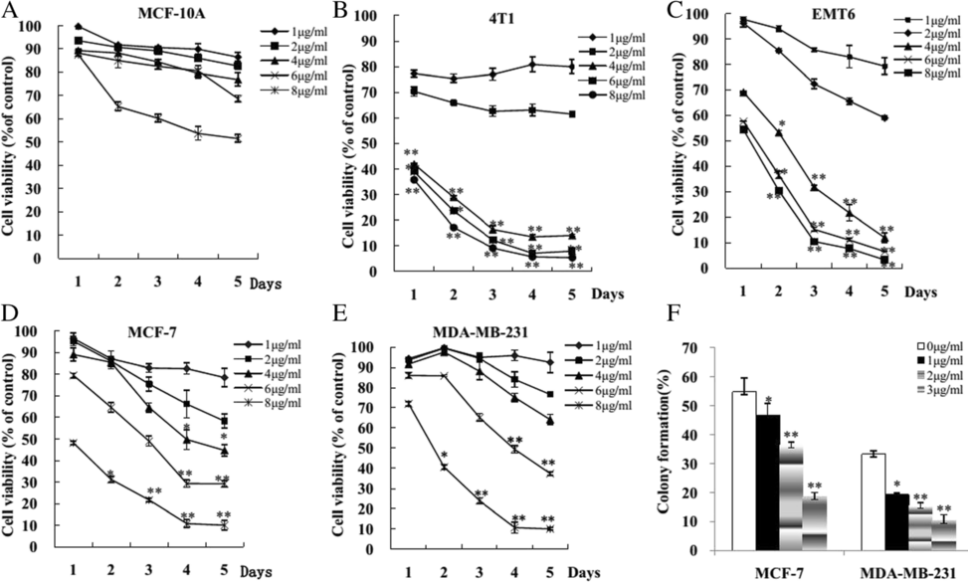
Morusin inhibited breast cancer cell growth and colony formation in vitro. **a** Human normal mammary epithelial cells (MCF-10A). **b** Murine breast cancer cells (4 T1). **c** Murine breast cancer cells (EMT6). **d** Human breast cancer cells (MCF-7). **e** Human breast cancer cells (MDA-MB-231). **f** Morusin inhibited human breast cancer cell colony formation. *P < 0.05, **P < 0.01. Three independent experiments were performed

The proliferation inhibition effect of morusin on human normal mammary epithelial cells (MCF-10A) and breast cancer cells is shown in Fig. 1. Morusin exhibited a dose- and time-dependent inhibitory effect on murine and human breast cancer cells (p < 0.01). IC_50_ was 9.48 μg/ml for normal mammary epithelial cells (MCF-10A); 2.03 and 1.87 μg/ml for murine breast cancer cells (4 T1 and EMT6); and 2.71 and 3.86 μg/ml for human breast cancer cells (MCF-7 and MDA-MB-231), respectively, the maximal inhibition of cell growth (>80 %) was obtained at 8 μg/ml (Fig. 1b, c, d, e).

### Morusin inhibits breast cancer cell colony formation

Colony formation rates of untreated MCF-7 and MDA-MB-231breast cancer cells were 54.83 ± 4.75 and 33.33 ± 1.04 %, respectively. After treatment with 1, 2, and 3 μg/ml of morusin, Colony formation rates of MCF-7 cells were 46.67 ± 4.04, 36.50 ± 3.50 and 18.50 ± 1.50 %, respectively; the rates of MDA-MB-23 cells were 19.33 ± 0.58, 15.50 ± 0.87 and 10.33 ± 1.10 %, respectively. Dose-dependent colony-forming inhibitory effect was observed in both MCF-7 and MDA-MB-231breast cancer cells (Fig. 1f).

### Apoptotic cell detection by DAPI staining

**Fig. 2:**
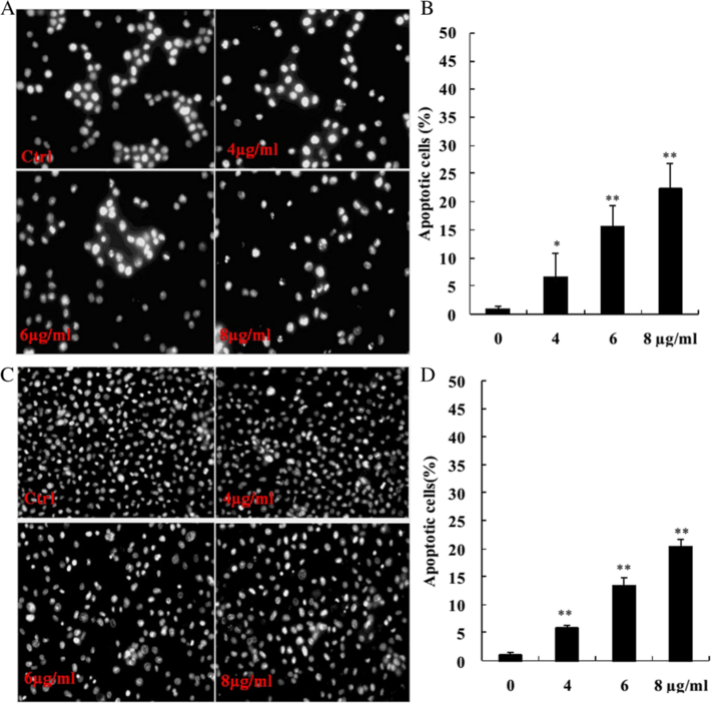
DAPI stained apoptotic cells in morusin treated MCF-7 and MDA-MB-231 breast cancer cells. **a** DAPI stained apoptotic cells in morusin treated MCF-7 cells. **b** The histogram shows that there was significant increase of AO/EB stained apoptotic cells in morusin treated MCF-7 cells. **c** DAPI stained apoptotic cells in morusin treated MDA-MB-231 cells. **d** The histogram shows that there was significant increase of DAPI stained apoptotic cells in morusin treated MDA-MB-231 cells. *P < 0.05, **P < 0.01. Three independent experiments were performed

The cells with condensed chromatin and stained bright with DAPI morphologically were apoptotic cells. Apoptotic cells in the untreated human breast cancer cells MCF-7 and MDA-MB-231 were 1.11 ± 0.37 % and 1.12 ± 0.47 %, respectively. After treatment with 4, 6, 8 μg/ml of morusin for 48 h, the apoptotic cells in breast cancer cells, MCF-7 were 6.7 ± 4.09, 15.70 ± 3.63, and 22.50 ± 4. 3 %, respectively; and in MDA-MB-231 cells, the apoptotic cells were 5.9 ± 0.51, 13.5 ± 1.4, 20.5 ± 1.3 %, respectively. The apoptotic cells in morusin treated breast cancer cells were increased significantly in a dose-dependent manner (*p* < 0.01, Fig. 2).

### Apoptotic cell detection by FCM

**Fig. 3:**
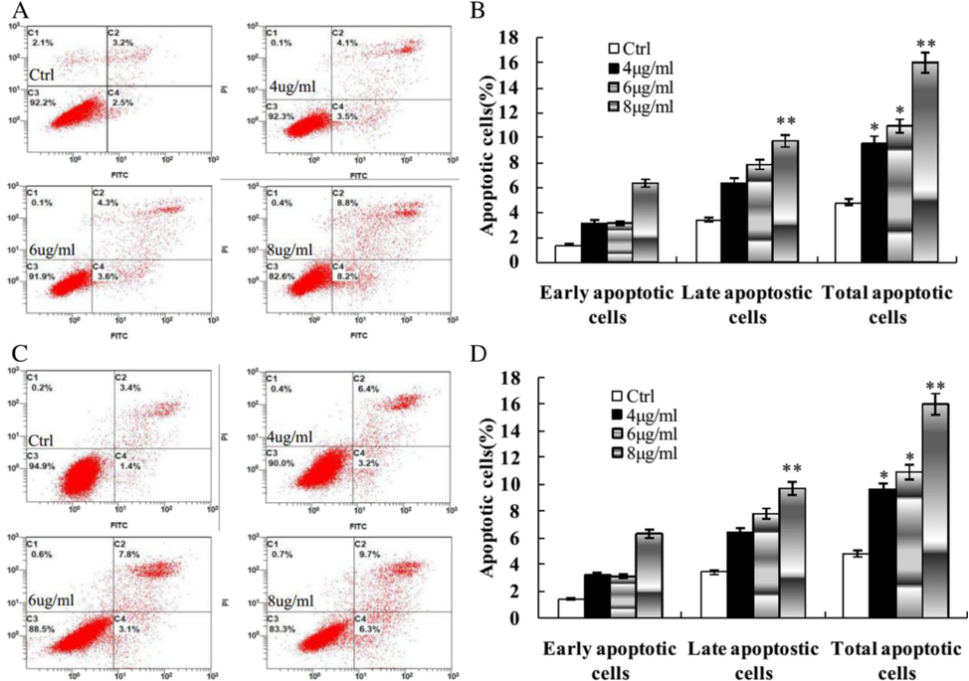
Annexin V-FITC stained apoptotic cells. **a** Morusin induces apoptosis of MCF-7 cells. **b** The histogram shows the significant increase of apoptotic cells in MCF-7 cells after treatment with morusin in a dose dependent manner. **c** Morusin induces apoptosis of MDA-MB-231 cells. **d** The histogram shows the significant increase of apoptotic cells in MDA-MB-231cells after treatment with morusin in a dose dependent manner. *P < 0.05, **P < 0.01. Three independent experiments were performed

Annexin-V-FITC/PI double staining assay and FCM analyses showed that apoptotic cells in the untreated human breast cancer cells MCF-7 and MDA-MB-231 were 4.70.11 ± 1.41 and 3.90 ± 1.27 %, respectively, after treatment with 4, 6, 8 μg/ml of morusin for 48 h, the apoptotic cells in the treated cancer cells increased significantly (*p* < 0.01, Fig. 3), in a dose-dependent manner.

### Flow cytometric analysis of cell cycle distribution

**Fig. 4:**
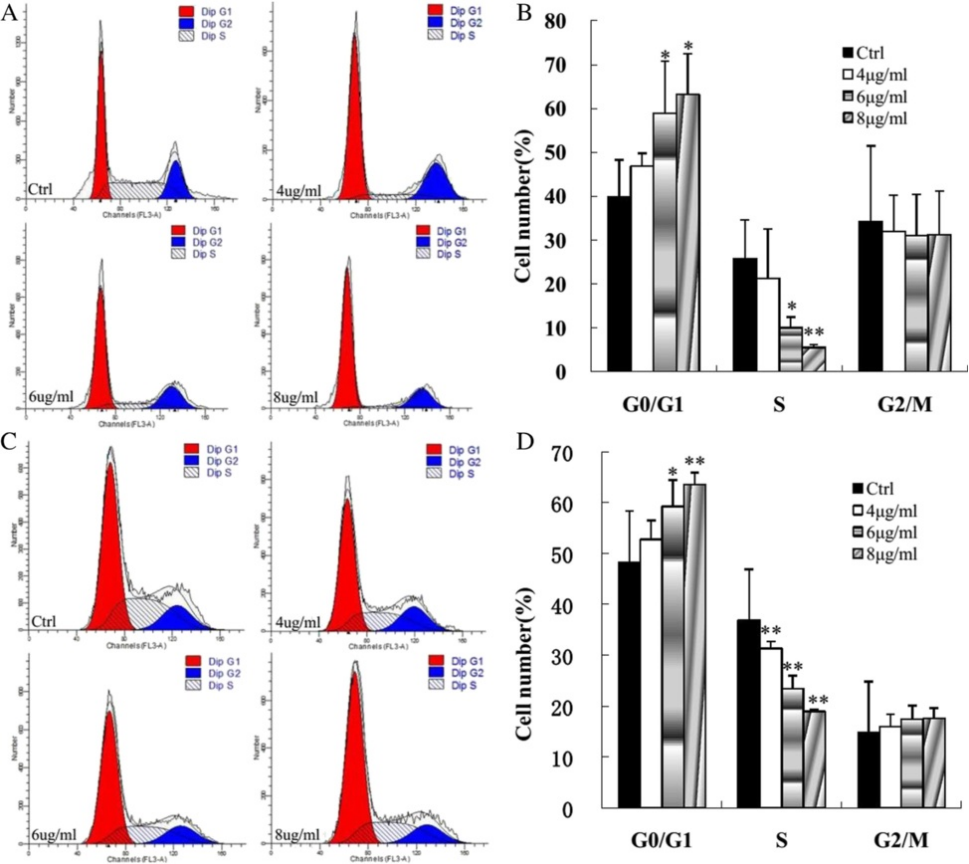
Morusin induced cell cycle arrest of human breast cancer cells. **a** Morusin induces cell cycle arrest of MCF-7 cells. **b** The histogram shows the significant increase of cell population in G_0_/G_1_ phase and decrease in S phase in a dose dependent manner in morusin treated MCF-7 cells **c** Morusin induces cell cycle arrest of MDA-MB-231 cells. **d** The histogram shows the significant increase of cell population in G_0_/G_1_ phase and decrease in S phase in a dose dependent manner in morusin treated MDA-MB-231 cells. *P < 0.05, **P < 0.01. Three independent experiments were performed

Cell cycle analysis was performed in MCF-7 and MDA-MB-231 cells after morusin treatment. Morusin treatment increased the cell population in G_0_/G_1_ phase and decreased the cell population in S phase in a dose dependent manner in both MCF-7 and MDA-MB-231cancer cells (Fig. 4).

### Morusin inhibited breast growth in vivo

**Fig. 5:**
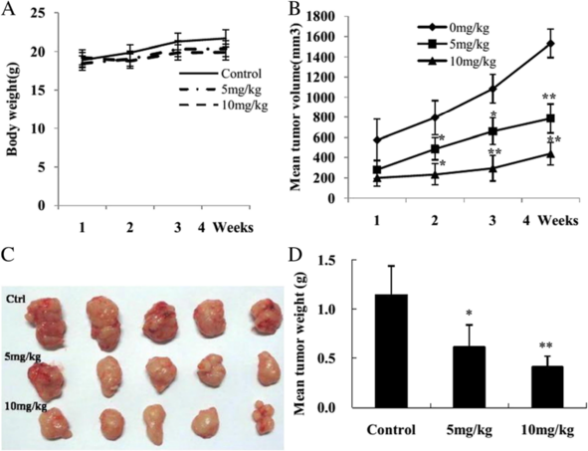
Morusin inhibited human breast cancer growth initiated from MCF-7 breast cancer cells in vivo. **a** Body weight changes of each group after morusin treatment. **b** Tumor volume changes of each group after morusin treatment. **c** Tumor masses of each group. **d** The histogram shows that there was a significant difference of mean tumor weight of each morusin treated group compared with the control. *P < 0.05, **P < 0.01

The result of tumor growth inhibition of morusin on human breast cancer generated from MCF-7 cells is shown in Fig. 5. To evaluate the growth inhibition effect of morusin on human breast in vivo, human breast cancer bearing-mice were injected with 5 and 10 mg/kg of morusin i. p., 3 times weekly for 4 weeks. Morusin retarded the growth of breast cancer significantly (*p* < 0.01, (Fig. 5b). Mean tumor weight of the control mice was 1.14 ± 0.30 g, and those of the mice administrated with 5 and 10 mg/kg of morusin were 0.61 ± 0.23 and 0.41 ± 0.10 g, respectively (Fig. 5c), tumor inhibitory rates were 46.5 % (*p* < 0.05), and 64.1 % (*p* < 0.01), respectively (Fig. 5d). No obvious evidence of toxicity was observed in treated animals by comparing the body weight increase (Fig. 5a), histopathological changes of major organs of both the control and treated animals.

### Adipocyte-like differentiation effect of morusin on breast cancer cells

**Fig. 6:**
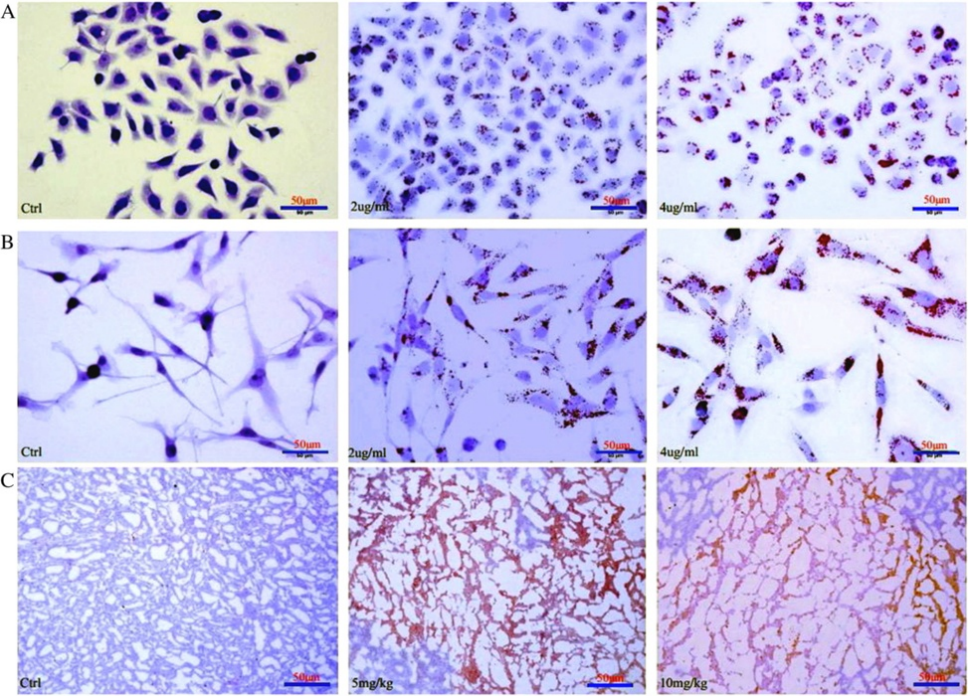
Adipogenic differentiation effect of morusin on human breast cancer cells. **a** Morusin induces adipocyte-like differentiation of MCF-7 breast cancer cells. **b** Morusin induces adipocyte-like differentiation of MDA-MB-231 breast cancer cells. **c** Morusin induces adipogenic differentiation of MCF-7 breast cancer cells in vivo

Fat vacuole accumulation was observed in morusin treated breast cancer cells and tumor tissues, staining of these cells with oil red O showed that these vacuoles were lipid droplets and located in the cytoplasm around the nucleus, lipid droplets were increased in a dose-dependent manner (Fig. 6).

### Morusin affected adipogenic and apoptotic protein expressions in vitro and in vivo

**Fig. 7:**
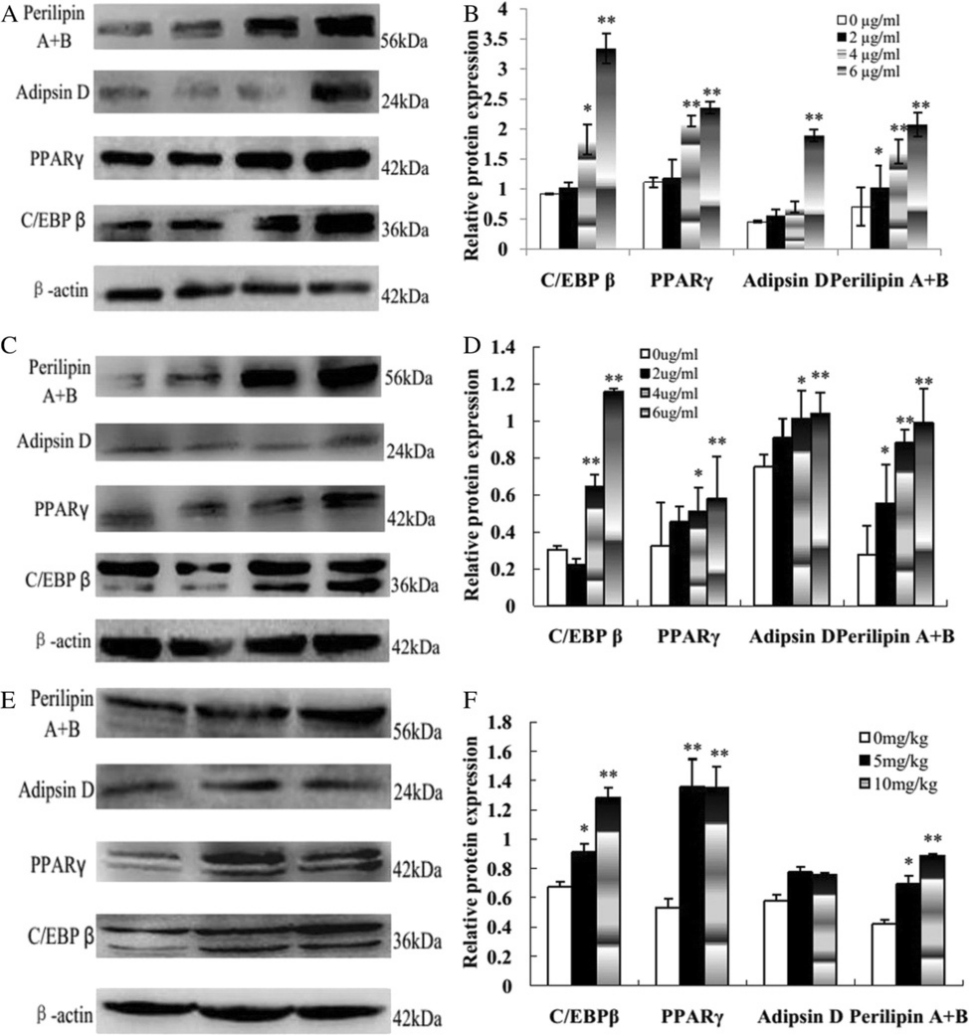
Adipogenic protein expressions in morusin treated human breast cancer cells and tumor tissues. **a** Western blot analysis of transcription factors C/EBP β and PPARγ and adipogenic protein expressions in morusin treated MCF-7 cells. **b** The histogram shows that there was significant increase of C/EBP β, PPARγ, adipsin D, and perilipin expressions in a dose dependent manner in morusin treated MCF-7 cells. **c** Western blot analysis of transcription factors C/EBP β and PPARγ and adipogenic protein expressions in morusin treated MDA-MB-231 cells. **d** The histogram shows that there was significant increase of C/EBP β, PPARγ, adipsin D, and perilipin expressions in a dose dependent manner in morusin treated MDA-MB-231 cells. **e** Western blot analysis of transcription factors C/EBP β and PPARγ and adipogenic protein expressions in morusin treated breast tumor tissues. **f** The histogram shows that there was significant increase of C/EBP β, PPARγ, adipsin D, and perilipin expressions in a dose dependent manner in morusin treated tumor tussues. *P < 0.05, **P < 0.01. Three independent experiments were performed

**Fig. 8:**
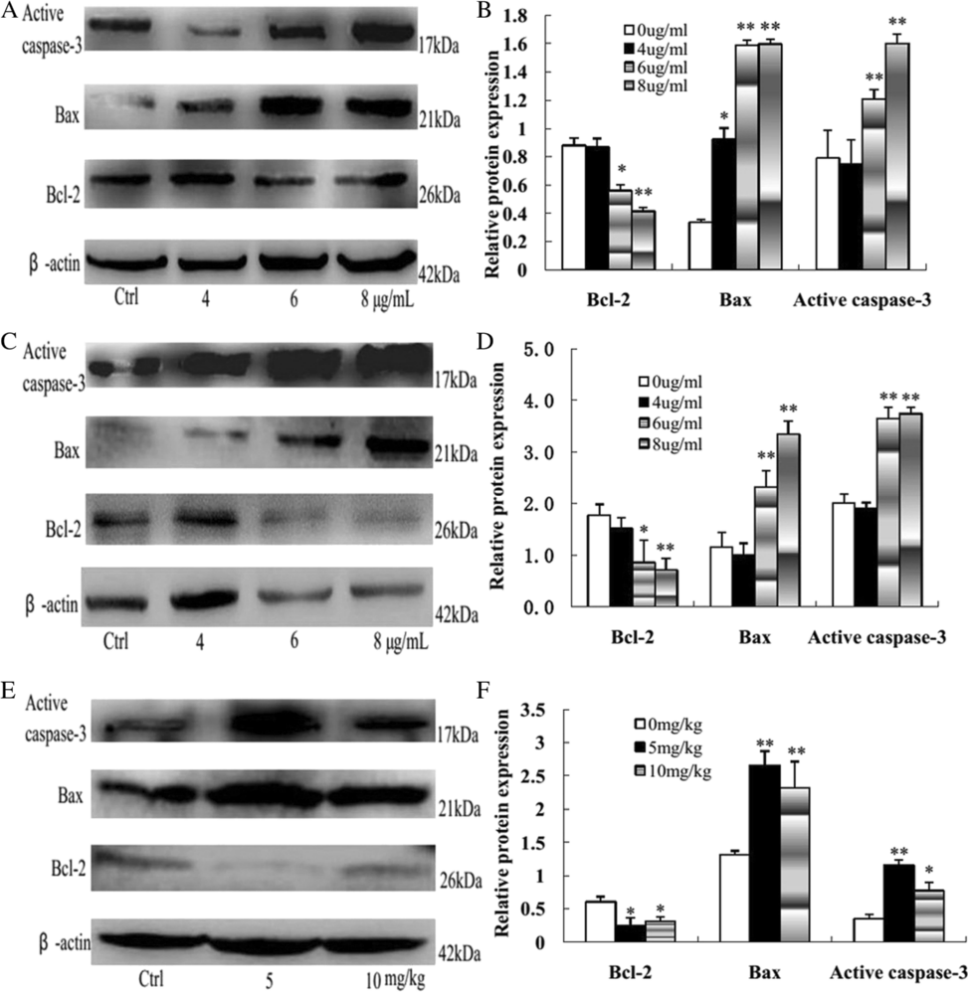
Apoptotic protein expressions in morusin treated human breast cancer cells and tumor tissues. **a** Western blot analysis of apoptotic protein expressions in morusin treated MCF-7 cells. **b** The histogram shows that there was significant increase of Bax and active caspaes-3 and decrease of Bcl-2 expressions in a dose dependent manner in morusin treated MCF-7 cells. **c** Western blot analysis of apoptotic protein expressions in morusin treated MDA-MB-231 cells. **d** The histogram shows that there was significant increase of Bax and active caspaes-3 and decrease of Bcl-2 expressions in a dose dependent manner in morusin treated MDA-MB-231 cells. **e** Western blot analysis of apoptotic protein expressions in morusin treated breast tumor tissues. **f** The histogram shows that there was significant increase of Bax and active caspaes-3 and decrease of Bcl-2 expressions in a dose dependent manner in morusin treated tumor tussues. *P < 0.05, **P < 0.01. Three independent experiments were performed

To explore the potential molecular mechanisms underlying the growth inhibition effect of morusin on human breast cancer cells in vitro and in vivo, transcription factors C/EBP β and PPARγ, adipogenic and apoptotic proteins in morusin treated breast cancer cells and tumor tissues were evaluated with Western blotting. Expressions of transcription factors C/EBP β and PPARγ, adipogenic proteins including adipsin D and perilipin, were increased significantly (Fig. 7, *p* < 0.05); In expressions of apoptotic proteins, Bcl-2 were decreased, Bax and active caspaes-3 were increased significantly (Fig. 8, *p* < 0.05) in a dose-dependent manner in both of morusin treated breast cancer cells and tumor tissues compared with the controls.

## Discussion

Breast cancer is most common malignant cancer, remains a leading cause of cancer-related death internationally. It is considered as a major public health problem around the world [1, 2, 28]. Its increasing incidence and mortality urgently needs identification and development of new and effective treatment options and chemopreventive strategies [8–11].

Flavonoids are present everywhere in nature, different flavonoids exhibited specific anticancer activities and could be purposely used both in cancer treatment as well as in chemoprevention [29], and some flavonoids are useful for prevention or treatment of breast cancer [14]. Prenylated flavonoids are a unique class of naturally occurring flavonoids that exist especially for the plant’s self-defensive function, the prenylation brings prenylated flavonoids with antibacterial, anti-inflammatory, antioxidant, cytotoxicity and estrogenic activities, thus, their bioactivities and action mechanisms need to be further investigated [30].

Morusin is a prenylated flavonoid isolated from the root bark of Morusaustralis and branch bark of Ramulus mori [15, 16], it can also be synthesized [17]. Existed studies showed morusin processes cytotoxicity against some human cancer cells in vitro [15–21] with little side effects [16, 17]. However, its anticancer efficiency and profile in vivo needs to be confirmed and its mechanism of activity remains to be elucidated [17–22].

In the present study, murine and human breast cancer cells, including ER+ breast cancer cells (MCF-7, ERα+) and TNBC cells (MDA-MB-231 and 4 T1) were treated with morusin, the cell proliferation of cancer cells were suppressed in dose and time-dependent manner (Fig. 1), the colony growth potential of cancer cells was inhibited in a dose-dependent manner (Fig. 1d, *p* < 0.01), and little cytotoxicity to normal mammary epithelial cells was noted (Fig. 1a).

Inducing apoptosis and cell cycle arrest are two main strategies for cancer treatment, many anticancer agents target rapidly cycling tumor cells, induce them cell cycle arrest and apoptotic death, and inhibit tumor growth in vivo [30–32].

After treatment with morusin, apoptotic cells were increased significantly in a dose-dependent manner (*p* < 0.01, Figs. 2 and 3), cell cycle arrest of morusin treated cancer cells was verified with flow cytometry analysis, the cell numbers in G_0_/G_1_ phases were increased and decreased in S phase significantly (*p* < 0.01, Fig. 4) in morusin treated cancer cells, which are the important hallmarks of apoptosis [33–35].

In vivo experiment,morusin supressed the growth of human breast cancer in vivo, reduced the volume and weight of tumor masses significantly (*p* < 0.01, Fig. 5), without evident toxicity [16, 22]. It is suggested that morusin might have potential anticancer activity on human breast cancer in vivo.

Furthermore, when breast cancer cells were exposed to low concentrations of morusin (2–6 μg/ml) in vitro, most cells survived and differentiated into adipocyte-like cells. In addition, focal adipogenetic differentiation was detected in morusin treated breast cancer tissues (Fig. 6).

To explore the molecular mechanisms of the growth inhibition effect of morusin on human breast cancer cells in vitro and in vivo, transcription factors C/EBP β and PPARγ, adipogenic and apoptotic proteins in morusin treated cancer cells and tumor tissues were analyzed with Western blotting. After morusin treatment, transcription factors C/EBP β and PPARγ, adipogenic proteins including adipsin D and perilipin were increased significantly (*p* < 0.05, Fig. 7) both in morusin treated cancer cells and tumor tissues, which were widely used molecular markers of adipocyte differentiation [36–39]; In apoptotic protein expression, anti-apoptosis protein, Bcl-2 were decreased, pro-apoptosis protein, bax and active caspaes-3 were increased significantly (*p* < 0.05, Fig. 8), which play important roles in the regulation of mitochondrial-mediated apoptosis [40, 41].

CCAAT-enhancer binding protein β (C/EBPβ) plays a pivotal role in terminal adipocyte differentiation, it is induced early to transactivate the expression of two master transcription factors, C/EBPα and peroxisome proliferator-activated receptor γ (PPARγ) [42]. Besides, C/EBPβ is also a transcription factor necessary for growth and differentiation of mammary gland and plays a critical role in mammary gland development and breast cancer progression [43, 44]. Its expression decrease was associated with shorter overall survival of breast cancer patients and increase of lung metastasis of mouse breast cancer cells [44, 45]. Herein, morusin might act as an agonist of C/EBPβ and PPARγ, upregulate expressions of C/EBPβ and PPARγ, activated the cascade of adipogenic differentiation of breast cancer cells.

Moreover, PPARγ activation is associated with differentiation, proliferation inhibition of the normal and malignant cells [46] and reversal of malignant phenotype of breast cancer cells [47]. It plays potential roles in the apoptosis of many types of cancer cells; its expression was increased simultaneously when apoptosis occurred [47–51].

In this experimental study, morusin treatment forced breast cancer cells differentiated into adipocyte-like cells, a lot of lipid droplets were accumulated increasingly in these adipogenic differentiating cells, unlimited accumulation of lipid droplets in the differentiating cancer cells results in apoptotic cell death of the differentiated cancer cells due to lipoptosis [52, 53]. Lipoptosis induction might be a novel approach for cancer therapeutic strategy, but no agents, which directly induce lipoapoptosis of cancer cells, have thus far been identified [52–54].

Combined with the findings in the present study, it could be assumed that morusin might be an effective agent on inducing adipogenic differentiation and lipoapoptosis of breast cancer cells through modulating the pathways of adipogenic differentiation and apoptosis or lipoapoptosis.

## Conclusions

In summary, morusin has the potential to inhibit human breast cancer cells growth in vitro and in vivo through C/EBPβ and PPARγ mediated adipogenic differentiation and lipoapoptosis induction, it might serve as a novel therapeutic agent for the treatment and/or prevention of human breast cancer, including ER+ breast cancer and TNBC, and need to be investigated further.
